# Mitochondrial ion channels as targets for cardioprotection

**DOI:** 10.1111/jcmm.15341

**Published:** 2020-06-03

**Authors:** Derek J. Hausenloy, Rainer Schulz, Henrique Girao, Brenda R. Kwak, Diego De Stefani, Rosario Rizzuto, Paolo Bernardi, Fabio Di Lisa

**Affiliations:** ^1^ Cardiovascular & Metabolic Disorders Program Duke‐National University of Singapore Medical School Singapore Singapore; ^2^ National Heart Research Institute Singapore National Heart Centre Singapore Singapore; ^3^ Yong Loo Lin School of Medicine National University Singapore Singapore Singapore; ^4^ The Hatter Cardiovascular Institute University College London London UK; ^5^ Cardiovascular Research Center College of Medical and Health Sciences Asia University Taichung City Taiwan; ^6^ Institute of Physiology Justus‐Liebig University Giessen Giessen Germany; ^7^ Coimbra Institute for Clinical and Biomedical Research (iCBR), Faculty of Medicine University of Coimbra Coimbra Portugal; ^8^ Center for Innovative Biomedicine and Biotechnology (CIBB) University of Coimbra Coimbra Portugal; ^9^ Clinical Academic Centre of Coimbra CACC Coimbra Portugal; ^10^ Department of Pathology and Immunology University of Geneva Geneva Switzerland; ^11^ Department of Biomedical Sciences University of Padova Padova Italy; ^12^ CNR Neuroscience Institute Padova Italy

**Keywords:** acute ischaemia/reperfusion injury, cardioprotection, Mitochondria, mitochondrial permeability transition pore

## Abstract

Acute myocardial infarction (AMI) and the heart failure (HF) that often result remain the leading causes of death and disability worldwide. As such, new therapeutic targets need to be discovered to protect the myocardium against acute ischaemia/reperfusion (I/R) injury in order to reduce myocardial infarct (MI) size, preserve left ventricular function and prevent the onset of HF. Mitochondrial dysfunction during acute I/R injury is a critical determinant of cell death following AMI, and therefore, ion channels in the inner mitochondrial membrane, which are known to influence cell death and survival, provide potential therapeutic targets for cardioprotection. In this article, we review the role of mitochondrial ion channels, which are known to modulate susceptibility to acute myocardial I/R injury, and we explore their potential roles as therapeutic targets for reducing MI size and preventing HF following AMI.

## 1 INTRODUCTION

Acute myocardial infarction (AMI) and the heart failure (HF) that often result remain the leading causes of death and disability worldwide. As such, new therapeutic targets are needed to protect the myocardium against the detrimental effects of acute ischaemia/reperfusion (I/R) injury in order to reduce myocardial infarct (MI) size, preserve left ventricular (LV) function and prevent the onset of HF. Mitochondrial dysfunction during acute I/R injury is a critical determinant of cell death following AMI, and therefore, ion channels in the inner mitochondrial membrane (IMM), which are known to influence cell death and survival, provide potential therapeutic targets for cardioprotection. In this article, we review the role of mitochondrial ion channels (including the mitochondrial permeability transition pore [PTP], mitochondrial calcium uniporter, mitochondrial potassium channels, connexin‐43 and mitochondrial uncoupling proteins [UCPs]), which have been shown to modulate susceptibility to acute myocardial I/R injury, and we highlight their potential roles as therapeutic targets for reducing MI size and preventing HF following AMI. However, recent attempts to translate cardioprotective strategies that target some of these mitochondrial ion channels have been hugely disappointing, and the translational issues facing mitochondrial protection are also discussed in this article, and in other articles of this special issue of JCMM.

## 2 THE MITOCHONDRIAL PTP

The mitochondrial permeability transition (PT) defines a sudden increase in the permeability of the IMM to solutes with molecular masses up to 1500 Da. This process is attributed to the opening of a voltage‐ and Ca^2+^‐dependent high‐conductance channel known as the mitochondrial PT pore (PTP).^1^ Besides Ca^2+^, PTP opening is favoured by reactive oxygen species (ROS), and a decrease in mitochondrial membrane potential (ΔΨ_m_), and it is antagonized by Mg^2+^, adenine nucleotides and especially matrix pH values <7.^1^, ^2^ The immediate and obligatory consequence of PTP opening is the dissipation of the proton gradient across the IMM that, in an uncoupling‐like effect, hampers adenosine triphosphate (ATP) synthesis and favours ATP hydrolysis. As the proton gradient is also utilized for mitochondrial Ca^2+^ uptake, PTP opening, which is stimulated by matrix Ca^2+^ accumulation, inhibits further Ca^2+^ entry while causing its release. This process appears to represent a physiological aspect of PT that might act as a pathway to avoid a potentially detrimental rise in intramitochondrial Ca^2+^ levels.^3^ Indeed, pharmacological and genetic inhibition of PTP opening results in increased matrix [Ca^2+^].^4^, ^5^ It is worth pointing out that PTP opening and the consequent matrix swelling are reversible, as initially documented in isolated mitochondria.^6^ In isolated cells, openings of short duration (ie, <1 sec) are apparently not associated with changes in ΔΨ_m_, simply because intracellular redistribution of fluorescent probes used for monitoring changes in mitochondrial membrane potential is slower. Nevertheless, changes in mitochondrial calcein fluorescence provided evidence that in untreated cells, transient PTP openings occur without affecting cell viability.^7^ However, at present, especially in intact hearts or in vivo, specific interventions are not available to distinguish between short‐ and long‐duration openings. This methodological limitation does not exclude the hypothesis that transient PTP openings contribute to cardioprotection induced by ischaemic pre‐conditioning (IPC).^8^

This limited, possibly problematic approach, results from the combination of the cardioprotective effects elicited by drugs interacting with cyclophilin D (CyPD), such as cyclosporin A (CsA),^1^, ^9^ with the lack of definitive knowledge on the molecular identity of the PTP.^10^ In this latter respect, genetic studies have ruled out the possibility that the PTP is formed by proteins located to the IMM, such as the adenine nucleotide translocase (ANT) and the phosphate carrier, or to the outer mitochondrial membrane, such as the voltage‐dependent anion channel (VDAC) or the benzodiazepine receptor or TSPO.^11^, ^12^, ^13^ More recently, data from different laboratories concur in suggesting that F‐ATP synthase, namely the enzyme that couples the proton gradient with ATP synthesis (or hydrolysis), is directly involved in PTP formation.^10^, ^14^ Two major models are now being validated by multiple approaches. In one model, the *c* subunit is suggested to act as a pore, although several features of the PTP, especially the Ca^2+^ sensitivity, are not displayed by patch clamp studies of purified *c* subunits.^15^ Conversely, all known features of the PTP are observed in electrophysiological studies carried out in purified F‐ATP synthase dimers, as opposed to the lack of response to Ca^2+^ displayed by monomers.^16^, ^17^ Further support to the role of F‐ATP synthase in PTP formation is provided by showing that major features of the PTP, such as Ca^2+^‐ and voltage dependence, as well as pH modulation, are selectively lost in mutants generated by means of site‐directed mutagenesis.^10^ In particular, the mutation of a single histidyl residue in the oligomycin sensitivity conferral protein subunit abolishes the pH‐dependence making the H112Q mutant more susceptible to cell death induced by simulated ischaemia. This finding supports the notion that PTP opening is inhibited by acidosis during ischaemia.^18^

**FIGURE 1:**
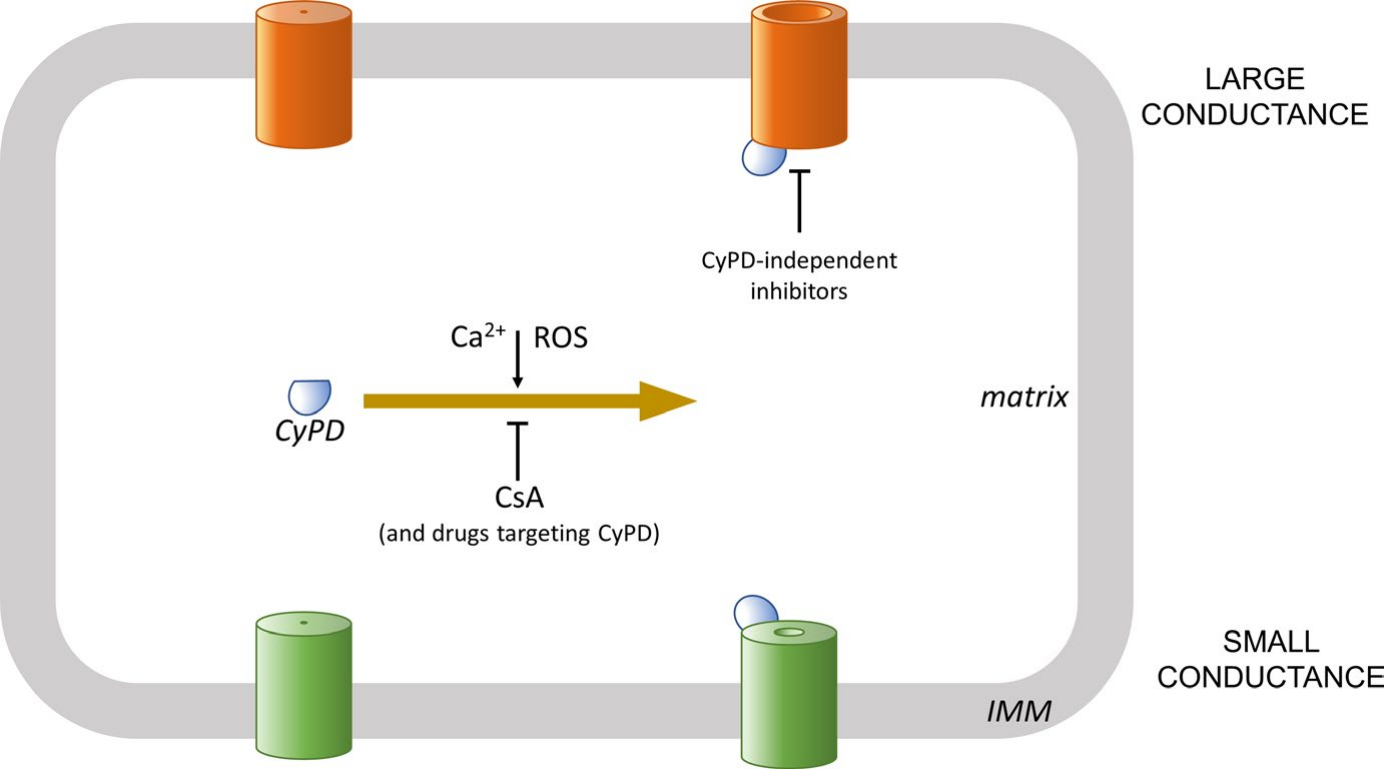
Current hypotheses on PTP opening. As illustrated in the upper part of figure, an increase in matrix levels of Ca^2+^ and/or ROS promotes opening of the large conductance PTP. This process is contributed by binding of CyPD to the PTP. Therefore, drugs, such as CsA, or genetic interventions targeting CyPD decrease, but do not abolish the open probability of the PTP. More recently, Ca^2+^‐induced PTP opening with small conductance has been reported that can still be inhibited by CsA. Although the proteins involved have not yet been conclusively identified, as discussed in Section 2, available information supports the involvement of F‐ATP synthase in forming the large conductance PTP. The small conductance could be due to other proteins, such as ANT, explaining the use of a different colour in lower part of the scheme. However, different conformations of the same protein might form pores with increasing sizes. CsA, cyclosporin A; CyPD, cyclophylin D; IMM, inner mitochondrial membrane; ROS, reactive oxygen species

The formation of the PTP from F‐ATP synthase has been questioned by deleting various subunits of this enzyme. Those deletions prevented assembly of functional F‐ATPase, yet Ca^2+^ ‐dependent Ca^2+^ release apparently remained. However, Ca^2+^‐induced swelling occurred after an abnormal lag with a slower rate and to a reduced extent. These features are not characteristic of the large conductance PTP, but they could suggest opening of a smaller channel (Figure 1). Interestingly, mutants lacking *c* subunits display small conductance properties inhibited not only by CsA, but also by the selective ANT inhibitor bongkrekic acid.^19^ Whereas this finding might explain previous and recent results on ANT contribution to PTP formation,^20^, ^21^ mitochondria appear to contain pores with different conductances formed by conformational changes of various proteins, and F‐ATP synthase is required for formation of the large conductance PTP. Whereas opening of the large conductance pore is most likely to contribute to pathological processes, small conductance PTP channels could play physiological roles, including self‐defence mechanisms. Besides testing this hypothesis by genetic approaches, for translational approaches pharmacological studies should also be implemented using PTP inhibitors that do not target CyPD. Recently, isoxazole and triazole compounds have been reported to afford a remarkable cardioprotective efficacy independent of CyPD.^22^ Besides identifying the molecular target(s), future studies should investigate whether these novel PTP inhibitors are devoid of the detrimental effects of a prolonged CyPD deletion or targeting, such as worsening of adverse LV remodelling in hypertrophied hearts.^5^

At variance from our current limited knowledge on possible physiological roles, a large body of evidence supports a pathological role for PTP opening,^1^, ^23^, ^24^, ^25^ especially in the case of the loss of cardiomyocyte viability associated with I/R injury.^26^, ^27^, ^28^, ^29^, ^30^, ^31^ The evidence supporting this hypothesis is indirect, as it is largely based upon the protective effects afforded by pharmacological or genetic approaches that prevent PTP opening. Although many small animal studies have shown that the administration of CsA at the time of reperfusion to target PTP opening, reduces MI size (reviewed in 32), the studies in large animal pig AMI models have been mixed.^33^, ^34^ Endogenous cardioprotective strategies such as IPC and ischaemic post‐conditioning have also been shown to mediate their protective effects by inhibiting PTP opening at reperfusion.^35^, ^36^

Other than in vitro techniques for detecting PTP opening in isolated mitochondria or cells,^37^ methods or biomarkers for detection of PTP opening in vivo are not available. Thus, not only the precise time and the mechanisms driving PTP opening have hardly been characterized in vivo, but also the PTP contribution to any given process still relies solely on interventions that in most cases target the PTP modulator CyPD and not the pore itself.^1^, ^38^ This indirect approach has serious shortcomings that complicate the possibility to translate experimental data into clinical settings, as well as the interpretation of data obtained in clinical trials,^38^ as discussed in Section 8.

In summary, whereas PTP opening is of crucial relevance in acute myocardial I/R injury, the conclusive elucidation of its molecular nature is necessary to solve the current uncertainties on functional modalities and their involvement in pathophysiology. This information will allow the development of direct and specific drugs to be tested in clinical trials for the proper re‐evaluation of PTP inhibition in human cardioprotection.

## 3 THE MITOCHONDRIAL CALCIUM UNIPORTER

Apart from many crucial roles played in excitation‐contracting coupling and signalling pathways in the cytosol,^39^, ^40^, ^41^ Ca^2+^ represents a pivotal factor in both mitochondrial physiology and mitochondria‐dependent pathological processes.^42^, ^43^, ^44^, ^45^, ^46^, ^47^ Within the matrix, Ca^2+^ contributes to the activation of key enzymes in substrate oxidation that fuel the respiratory chain with reducing equivalents resulting in an increased ATP synthesis to match the increased demand dictated by a rise in cytosolic [Ca^2+^]. The contribution of mitochondrial Ca^2+^ uptake to cytosolic Ca^2+^ oscillations related to myocardial contractile activity remains a controversial issue.^48^ However, as mentioned in Section 2, a large rise in mitochondrial Ca^2+^ is a major factor in promoting PTP opening. Unfortunately, despite major advancements in techniques for monitoring Ca^2+^ levels in various cellular compartments, a threshold between physiological and pathological [Ca^2+^] cannot yet be established even in vitro. Nevertheless, influx and efflux pathways operate continuously to regulate the intramitochondrial (ie, matrix) [Ca^2+^] with respect to Ca^2+^ levels in the cytosol. The electrochemical gradient (∆µ_H_) across IMM generated by mitochondrial respiration drives both Ca^2+^ uptake and release. Indeed, the electrical component of ∆µ_H_, namely Dy, drives Ca^2+^ entry into the matrix by means of the mitochondrial calcium uniporter (MCU), whereas the chemical component of ∆µ_H,_ namely ∆pH, is utilized for mitochondrial Ca^2+^ release catalysed mostly by the mitochondrial Na^+^/Ca^2+^ exchanger (or more precisely, Na^+^/Ca^2+^ Li^+^‐permeable exchanger, abbreviated as mNCLX).^49^ In fact, mNCLX is functionally coupled with the mitochondrial Na^+^/H^+^ exchanger, so that eventually mitochondrial Ca^2+^ release is paralleled by H^+^ uptake. In addition, PTP opening might represent a fast pathway for mitochondrial Ca^2+^ release (see Section 2).

Our current understanding of mitochondrial Ca^2+^ homoeostasis has been greatly advanced by the elucidation of the molecular nature of MCU and mNCX.^50^, ^51^ MCU is a 40 kD protein with two transmembrane domains separated by a short loop containing acidic residues that are critical for Ca^2+^ channelling function. The discovery of the MCU was preceded by the identification of the mitochondrial calcium uptake protein 1^52^ that acts as a gatekeeper preventing mitochondrial Ca^2+^ uptake at low cytosolic [Ca^2+^].^47^, ^53^, ^54^, ^55^ More recently other proteins have been identified that control MCU activity and are grouped under the term MCU complex (reviewed in 47, 53, 56). Therefore, the net mitochondrial Ca^2+^ uptake results from the balance between the activities of several proteins. This structural complexity hinders the development of genetic or pharmacological strategies aimed at controlling mitochondrial Ca^2+^ levels, as well as the interpretation of results from loss‐of‐function approaches. Indeed, this appears to be the case with studies investigating MCU role in acute myocardial I/R injury. As upon reperfusion the large increase in mitochondrial [Ca^2+^] occurring upon reperfusion favours PTP opening, MCU deletion should confer protection against I/R injury. However, mice models have generated controversial results.^57^ Although MCU deletion was embryonically lethal in a pure genetic background (ie, C57BL/6), mice devoid of MCU were generated in a mixed genetic background (ie, CD1). Not only was basal cardiac function not affected, but also, when hearts were isolated and subjected to I/R injury, infarct size was not different from WT littermates and CsA‐induced protection was abolished.^58^ Also lack of protection resulted from MCU down‐regulation obtained by means of transgenic expression of a dominant negative (MCU).^59^ Conversely, the specific deletion of cardiac MCU in adult mice elicited a significant protection against in vivo I/R injury.^60^, ^61^ The discrepancy between results in total body deletion and conditional knockout has not been resolved. Besides differences between in vitro and in vivo acute myocardial I/R injury models, timing and duration of MCU deletion are likely to explain the difference in sensitivity to I/R injury. The lack of MCU throughout development might trigger compensatory mechanisms involved in death pathways.^57^

In summary, whereas results from a prolonged MCU deletion would suggest that cardiomyocyte death can be obtained independently of mitochondrial Ca^2+^ overload and PTP opening, findings in conditional MCU KO mice lend support to the relevance of mitochondrial Ca^2+^ overload as a determining factor in acute myocardial I/R injury. This concept is further supported by genetic manipulations of mNCLX. Its deletion was shown to cause a severe mitochondrial Ca^2+^ overload resulting in lethal HF, whereas cardiac specific overexpression prevented I/R‐induced PTP opening and cardiomyocyte death.^62^ Drugs specifically targeting the MCU complex are necessary to validate or dispute the occurrence and the role of mitochondrial Ca^2+^ increase in I/R injury models in large animals and clinical settings.

## 4 THE MITOCHONDRIAL K_ATP_ CHANNEL

Several K^+^ channels have been described in mitochondria.^63^, ^64^, ^65^ However, due to its role in cardioprotection most of the attention has been focused on the mitochondrial ATP‐sensitive (ie, inhibited by physiological levels of ATP) K^+^ channel (mitoK_ATP_).^66^, ^67^, ^68^, ^69^, ^70^ The maintenance of matrix volume depends on the net balance between K^+^ uptake and release.^64^, ^71^ A K^+^ cycle across the IMM exists whereby the electrophoretic influx driven by Δѱ_m_ is balanced by the electroneutral K^+^/H^+^ antiporter.^71^ An uncontrolled increase in K^+^ influx, such as that induced by valinomycin, results in mitochondrial depolarization and large matrix swelling, so that K^+^ channels have to be tightly regulated. This is the case with mitoK_ATP_ that was originally described in liver mitoplasts.^72^ Its function was initially attributed to the regulation of mitochondrial volume^73^ with no precise role in pathophysiology in any organ, in contrast from various functions attributed to plasma membrane K_ATP_ channels.^74^, ^75^ The mitoK_ATP_ channels are hetero‐octameric complexes of 4 transmembrane inward‐rectifying K^+^ (KIR) channel subunits and four sulfonylurea receptor (SUR) subunits. Two KIR isoforms (KIR6.1/6.2) and 3 SUR isoforms (SUR1/2A/2B) have been described.^75^ For instance, in the heart the sarcolemmal K_ATP_ channel (surfaceK_ATP_) comprises KIR6.2 and SUR2A subunits.

As its initial discovery, the cardiac surfaceK_ATP_ has been suggested to be associated with cardioprotection.^76^ Activation occurs in response to ischaemia‐induced [ATP] decrease. The consequent shortening of action potential reduces Ca^2+^ entry and contractility favouring the maintenance of ATP levels and cell viability during acute I/R injury (reviewed in 77). This hypothesis appeared to be supported by reports showing that K_ATP_ openers mimicked the cardioprotective effects of IPC, with IPC‐induced protection being abolished by K_ATP_ antagonists.^69^ However, subsequent reports argued against a major surfaceK_ATP_ involvement by showing that: (a) K_ATP_ channel activators, including diazoxide (DZX), protected non‐contracting cardiomyocytes^78^; (b) surfaceK_ATP_ channel activity was hardly affected by DZX, whereas drugs specifically targeting the surfaceK_ATP_ channel were not protective.^78^, ^79^ On the other hand, DZX was shown to restore ATP‐inhibited K^+^ flux in isolated mitochondria^80^ that also displayed 5‐hydroxydecanoate (5HD) inhibition.^81^ Therefore, the attention shifted towards mitochondria to both elucidate mitoK_ATP_ structure and clarify the protective mechanisms triggered by its opening.

The fact that DZX and 5HD also affected mitochondrial processes other than the K_ATP_ channel^63^ renders the identification of its molecular identity especially important. Initial attempts provided evidence that electrophysiological properties of K^+^ channels can be observed in lipid bilayers containing partially purified mitochondrial proteins.^73^, ^82^ More recently, an unbiased proteomic approach carried out in cardiac IMM led to the identification of *Kir1.1*, another member of the *Kir* gene family, also known as the renal outer medullary K^+^ channel (ROMK).^68^ This protein has been attributed the role of mitoK_ATP_ channel based upon results obtained in cardiomyoblasts whereby ROMK overexpression protected against death stimuli. These results should be validated by in vivo approaches of loss‐ and gain of function. Of note, the pharmacologic properties of ROMK do not completely match those reported for mitoK_ATP_ and ROMK interaction with SUR components has not yet been demonstrated. Lately, mitoK_ATP_ identity has been attributed to a protein complex of the IMM that, similar to surfaceK_ATP_ channels, is composed of pore forming and ATP‐binding subunits, termed MITOK and MITO SUR, respectively.^70^ Whereas overexpression of only MITOK generated an excessive K^+^ influx resulting in large matrix swelling, the in vitro reconstitution of MITOK together with MITOSUR displayed the main electrophysiological and pharmacologic properties of mitoK_ATP_. In addition, MITOK deletion abolished DZX‐induced cardioprotection in isolated hearts.^70^ Regarding the protective mechanisms, mitoK_ATP_ opening causes a mild increase in mitochondrial levels of ROS that appears to be required for cardioprotection.^83^ Actually, a feedback loop has been proposed whereby ROS would result in PKC‐ε activation that would prolong mitoK_ATP_ activation and prevent PTP opening.^83^ This loop might also involve connexin43 (Cx43).^84^

In summary, whereas the involvement of mitoK_ATP_ in cardioprotection is generally accepted mostly based upon pharmacological evidence, the protective mechanisms are far from being conclusively defined. The recent identification of the molecular nature of the mitoK_ATP_ will provide the molecular tools to investigate its interaction with signalling pathways and determining factors of acute myocardial I/R injury, such as proteins involved in mitochondrial ROS formation, Ca^2+^ transport and PT.

## 5 THE MITOCHONDRIAL BKCa CHANNEL

In addition to the mitoK_ATP_ channel, there is also a large or “big” conductance calcium‐sensitive and voltage‐activated K^+^ channel (termed the BKCa channel) present in the IMM.^85^ A number of experimental studies have shown that opening of the BKCa channel, using the pharmacological agonist NS1619, reduced MI size in animal models of acute myocardial I/R injury and that this cardioprotective effect was abrogated in the presence of the BKCa channel antagonist paxilline.^85^, ^86^ In addition, mice overexpressing BKCa channel were found to be protected against acute I/R injury, confirming the cardioprotective role of this mitochondrial potassium channel. The mechanisms underlying this cardioprotective effect have been attributed to its effect on mitochondrial membrane depolarization, attenuation of mitochondrial Ca^2+^ overload,^87^ production of mitochondrial signalling ROS,^88^ and reduced oxidative stress at reperfusion.^89^

Interestingly, in common with the mitoK_ATP_ channel, it has also been shown that the infarct‐limiting effects of both classical and delayed IPC,^86^, ^90^ ischaemic post‐conditioning (IPost),^91^ and limb remote ischaemic conditioning (RIC),^92^, ^93^ were abrogated in the presence of a pharmacological BKCa blocker, suggesting that opening of the BKCa channel is also required for ischaemic conditioning cardioprotection. Furthermore, it has been shown that protein kinase A (PKA), which is known to be activated by IPC, can increase opening of the BKCa channel, providing a potential link between IPC and BKCa channel opening.^87^

In summary, in a similar manner to the mitoK_ATP_ channel, the opening of the mitochondrial BKCa is cardioprotective and contributes to ischaemic conditioning protection. How the two mitochondrial potassium channels interaction to confer cardioprotection needs further investigation.

## 6 CONNEXIN43

**FIGURE 2:**
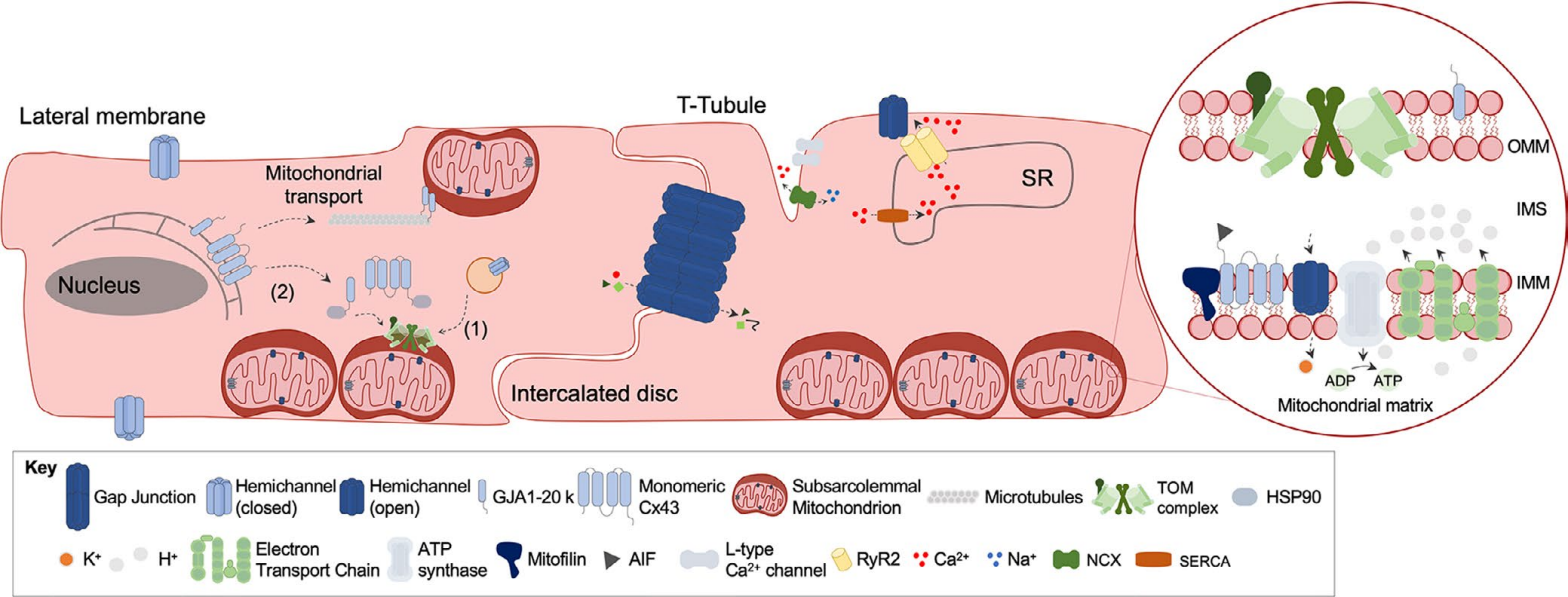
Mitochondrial Cx43 in cardiomyocytes. In cardiomyocytes, the accumulation of Cx43 in mitochondria occurs primarily in the inner membrane of subsarcolemmal mitochondria, where the close proximity to the plasma membrane and internalized annular gap junctions likely favour the transfer of Cx43 to the mitochondria. At mitochondria, Cx43 interacts with apoptosis‐inducing factor, beta‐subunit of the electron‐transfer protein, ATP synthase alpha and beta, cytochrome c oxidase subunit 4, voltage‐dependent anion channel protein 1, mitochondrial stress protein 70, mitofilin and subunits of the Kir6.1 potassium channels. Cx43‐hemichannels can also interact with ryanodine receptors at the sarcoplasmic reticulum. Besides the full length, a shorter NT‐truncated form of Cx43, the GJA1‐20 kD, is present in the outer mitochondrial membrane of cardiomyocytes, where it mediates the microtubule‐dependent mitochondrial transport

Cx43 is a protein well known for the formation of gap junctions. It is the main connexin isoform expressed in cardiomyocytes, but it is also found in other cell types of the heart such as endothelial cells and fibroblasts (for review, see ^94^, ^95^). Six Cx43 molecules assemble into hemichannels, and gap junctions are formed by docking of hemichannels from adjacent cells, which connect the cytoplasm of cells. Gap junctions are regulated by post‐translational modifications of Cx43, amongst them phosphorylation mediated by several protein kinases (for review, see ^95^, ^96^). In addition to its localization at the plasma membrane, Cx43 is also detected at other cellular compartments including the nucleus,^97^, ^98^ subsarcolemmal mitochondria,^99^, ^100^, ^101^, ^102^, ^103^ and exosomes,^104^, ^105^ which attribute to Cx43 other biological functions that extend beyond intercellular communication (for review, see ^105^, ^106^). Interestingly, gap junctions form intimate associations with mitochondria, and the contact between annular gap junction vesicles and mitochondria may facilitate Cx43 delivery to the mitochondria (Figure 2).^107^

Within mitochondria, Cx43 regulates central functions—all of them being important for I/R injury and protection from it—including oxygen consumption and ATP production,^108^ ROS formation,^109^, ^110^ uptake of K^+^ ^111^ and Ca^2+^,^112^ PTP opening^112^, ^113^ and apoptosis.^114^ The amount of mitochondrial Cx43 increases with ischaemia,^115^ and also rises by treatment with DZX or fibroblast growth factor 2, compounds known to reduce irreversible I/R injury.^100^, ^116^ Despite these above‐described functions of mitochondrial Cx43, the exact contribution of the protein to mitochondrial function in I/R injury and cardioprotection remains unclear. Cx43 interacts with a number of other proteins and the analysis of the interactome of whole cell Cx43 under normoxic conditions and after myocardial I/R injury by mass spectrometry identified proteins involved in metabolism, signalling, trafficking, but also in gap junction‐independent processes.^117^ Mass spectrometry analysis of native immunoprecipitated mitochondrial extracts showed that Cx43 interacts with the apoptosis‐inducing factor and the beta‐subunit of the electron‐transfer protein,^118^ the F‐ATP synthase α and β subunits, cytochrome c oxidase subunit 4, VDAC protein 1, mitochondrial stress protein 70 and mitofilin,^119^ a protein important for cristae structure and morphology.^120^ Mitochondrial Cx43 also interacts with subunits of the Kir6.1 potassium channels^121^ and it regulates the expression of mitochondrial nitric oxide synthase isoforms and thereby the mitochondrial nitric oxide formation.^122^ The importance of such protein‐protein interactions for mitochondrial function and I/R injury requires further investigation. The presence of Cx43 at the mitochondria is also important to regulate Ca^2+^ homoeostasis.^112^

A contribution of Cx43 to the cardioprotection by IPC is shown in heterozygous Cx43‐deficient mice,^123^ which have no longer MI size reduction after a classical IPC protocol.^123^ The first evidence that mitochondrial Cx43 is involved in such protection came from a study in isolated cardiomyocytes—apparently lacking gap junctions—in which cytoprotection by pre‐conditioning was achieved in wild‐type, but not in Cx43‐deficient cells.^124^ Inhibition of the mitochondrial import of Cx43 abolished MI size reduction by pharmacological pre‐conditioning.^125^ Although mitochondrial Cx43 increased after I/R, such an increase was absent in hearts undergoing a IPC protocol,^126^ and the phosphorylation status of mitochondrial Cx43 was preserved.^127^

Although isolated mitochondria can be protected by IPC, such beneficial effects depend on the presence of Cx43.^128^ The mechanism by which Cx43 contributes to cardioprotection by IPC seems to involve ROS signalling.^84^ In addition, S‐nitrosylation—a post‐translational modification of general importance for cardioprotective signalling—involves mitochondrial Cx43.^129^ Studies on the role of mitochondrial Cx43 in pre‐conditioning have recently been reviewed.^129^

Data on the contribution of mitochondrial Cx43 to RIC or IPost are limited. Although mitochondrial Cx43 increases after I/R, such an increase is absent in hearts undergoing an IPost protocol.^126^ Depending on the species, IPost is associated with either decreased^126^ or increased^130^ phosphorylation of Cx43. In H9C2 cells, hypoxic post‐conditioning enhanced the amount of mitochondrial Cx43 and the protein contributed to cytoprotection via decreasing ROS formation.^131^ In addition, phosphorylation of mitochondrial Cx43 is also involved in pharmacological post‐conditioning with sphingosine‐1‐phosphate (S1P).^132^ The S1P‐induced reduction of MI size after ex vivo I/R was lost in hearts of mice with a truncated C‐terminus of Cx43 (Cx43K258/KO) or in which the S368 is mutated to a non‐phosphorylatable alanine (Cx43S368A/S368A), illustrating the importance of this site in cardioprotection.^132^ Moreover, infusion of short peptides (αCT1 and αCT11), known to increase S368 phosphorylation of Cx43 in cardiomyocytes, preserved LV function when applied before or after ischaemia.^133^ However, it should be kept in mind that alterations in Cx43 expression/phosphorylation induced by ischaemic or pharmacological post‐conditioning may be involved but are likely not causal for such cardioprotection, as heterozygous Cx43‐deficient mice also show an effective infarct size reduction by IPost.^134^ Studies on the role of mitochondria in post‐conditioning have recently been reviewed.^129^

Connexin genes are commonly classified according to sequence homology and alphabetically divided into five subfamilies^135^ and the Cx43 gene is therefore called *GJA1*. Cx43 is subject to internal (alternative) translation, *that is* the translation of the coding region of *GJA1* mRNA produces not only full‐length Cx43 but at least 4 additional NT‐truncated forms with different molecular weight, of which the 20 kD form is the most abundant.^136^, ^137^, ^138^, ^139^, ^140^ Functionally, it was first shown that GJA1‐20k interacts with full‐length Cx43 thereby regulating its subcellular distribution and the formation of gap junctions. This mechanism appeared of particular importance to limit gap junction formation during epithelial‐mesenchymal transition.^136^, ^138^ In cardiomyocytes, GJA1‐20k stabilizes actin filaments to guide Cx43 delivery to the intercalated discs^140^ and it facilitates microtubule‐dependent mitochondrial transport important for mitochondrial network integrity during cellular stress.^139^

Recent studies showed an up‐regulation of GJA1‐20k upon hypoxia, ischaemia or I/R.^141^, ^142^, ^143^ GJA1‐20k targets to the outer mitochondrial membrane of cardiomyocytes.^143^ Furthermore, AAV9‐mediated gene transfer of GJA1‐20k in mouse hearts reduced I/R injury in vivo and ex vivo*.* Similar to what has been observed during IPC, GJA1‐20k promotes mitochondrial biogenesis and a protective mitochondrial phenotype, with a reduction of membrane potential, respiration and ROS production.^143^ Interestingly, Cx43 has also been observed in mitochondria of endothelial cells.^113^ However, the role of mitochondrial Cx43 in endothelial cells and its possible involvement in cardiac I/R remains to be investigated.

In summary, Cx43 not only forms gap junctions but is also present in mitochondria, where it is important for several aspects of mitochondrial function. Mitochondrial Cx43 is clearly involved in the cardioprotection by IPC.

## 7 MITOCHONDRIAL UCPs

Mitochondrial production of ATP to fuel cardiac contraction and relaxation is critically dependent on maintaining a large electrochemical gradient for protons across the mitochondrial inner membrane. Prior experimental studies have shown that mild chemical mitochondrial uncoupling can confer IPC‐like cardioprotection, suggesting that modifying mitochondrial respiratory function can mediate cardioprotection.^144^, ^145^ The mitochondrial UCPs are inner membrane carrier proteins that are able to induce proton leak and dissipate mitochondrial membrane potential, thereby reducing mitochondrial ROS production under conditions of stress, such as acute myocardial I/R injury (reviewed in 146). Overexpression of UCP1 has been shown to protect H9C2 heart‐derived cells, and the adult murine heart against acute I/R injury, by reducing oxidative stress at reperfusion.^147^, ^148^ Reperfusion has been shown to increase myocardial gene and protein expression of UCP2, and the overexpression of UCP2 has been reported to reduce susceptibility to acute myocardial I/R injury in the rat heart.^149^ IPC was shown to increase UCP2 expression, with siRNA knockdown of UCP2 abolishing IPC cardioprotection.^149^ Interestingly, delayed pre‐conditioning has also been reported to up‐regulate both UCP2 and UCP3 in the rat heart and reduce oxidative stress at reperfusion, with abrogation of UCP2 but not UCP3 blocking the cardioprotection elicited by delayed pre‐conditioning, suggesting a role of UCP2 in mediating endogenous cardioprotection due to classical and delayed IPC.^150^ A cardioprotective role for UCP3 has also been demonstrated, with mice deficient in UCP3 being more susceptible to acute myocardial I/R injury,^151^, ^152^ and cardiac arrhythmias.^152^ Interestingly, the cardioprotective effect of IPC was attenuated in these mice, suggesting a role for UCP3 as a mediator of IPC.^152^

In summary, overexpression of UCPs protects the myocardium against acute I/R injury, with UCP2 and UCP3 mediating the cardioprotection elicited by endogenous cardioprotection strategies such as IPC.

## 8 TRANSLATION OF MITOCHONDRIAL PROTECTION

Although a large number of experimental studies have demonstrated MI size reduction with pharmacological agents that target mitochondrial channels in experimental small and large animal models of myocardial I/R injury, the translation of mitochondrial protective strategies into the clinical setting for patient benefit has been hugely disappointing. Amongst these, the most promising mitochondrial target, had been the PTP, given the substantial experimental data demonstrating MI size reduction using CsA to target PTP opening at reperfusion—although not all studies had been positive.^34^, ^153^ Despite, an initial small proof‐of‐concept clinical study in AMI patients demonstrating a reduction in MI size with CsA administered at time of reperfusion compared to control,^29^, ^154^ two subsequent large randomized controlled trials, CIRCUS and CYCLE, failed to report any benefit with CsA on either MI size reduction or clinical outcomes.^155^, ^156^, ^157^ The reasons for this failure to translate PTP inhibition for clinical benefit in AMI patients are not clear but have been attributed to: insufficient delivery of CsA to ischaemic cardiomyocytes and the presence of factors which are known to confound cardioprotection, such as co‐morbidities (eg, age, diabetes), and co‐medications (eg, platelet P2Y12 inhibitors). Interestingly, experimental data has shown that PTP inhibition using CsA to reduce MI size was ineffective in pre‐diabetic Zucker obese rats compared to Zucker lean rats.^158^ CsA has also been tested as a cardioprotective agent in the setting of cardiac surgery, where it has been shown to reduce the extent of peri‐operative myocardial injury.^159^, ^160^ Other therapeutic strategies that target mitochondria to reduce MI size have also failed in AMI patients—these include elamipretide (a cell‐permeable peptide suggested to preserve the integrity of cardiolipin),^161^ and TRO40303 (suggested to inhibit the TSPO).^162^

In summary, despite there being substantial experimental data supporting the targeting of mitochondria as a therapeutic strategy to reduce MI size, the translation to the clinical setting for patient benefit has been disappointing. It may well be that other more potent PTP inhibitors that are CypD‐independent,^22^ may provide alternative cardioprotective strategies in future studies.

## 9 CONCLUSIONS

A number of mitochondrial ion channels in the IMM have been implicated as therapeutic targets for cardioprotection. The compounds used are summarized in Table 1. The mechanisms underlying the protective effects of these channels have not been fully elucidated and need further study. Of these channels, the mitochondrial PTP has been extensively investigated as a cardioprotective target in the pre‐clinical setting using CsA to inhibit PTP opening at reperfusion, but the results in the clinical setting of AMI have been disappointing. Recent advances in the molecular identification of some of these mitochondrial ion channels (eg, MCU, PTP, MitoK_ATP_) will be helpful in this regard and allow the design of new more specific modulators of these mitochondrial ion channels for translating cardioprotection into the clinical setting for patient benefit.

**TABLE 1:** Compounds that have been shown to modulate mitochondrial channels and have been tested for reduction of myocardial infarct size

Channel	Effect	Compound	Species	Ex vivo	In vivo	Notes	References
PTP	Inhibition	CsA	Mouse		x		163, 164
			Rat		x		165, 166
				x			30, 169
					x	No protection	153
			Rabbit		x		170, 171
			Pig		x		33
					x	No protection	175, 176
		Debio‐025	Mouse		x		177
		Sanglifehrin A	Mouse		x		178
			Rat	x			31
		NIM 811	Rabbit		x		172
		Compound 22	Rabbit		x		179
K_ATP_	Activation	Diazoxide	Rat	x			66
					x		180, 181
			Rabbit		x		183, 184
					x	No protection	187
BKCa	Activation	NS1619	Rat	x			188
			Rabbit	x			85
			Dog		x		86

## CONFLICTS OF INTERESTS

All authors have no conflicts or disclosures.

## AUTHOR CONTRIBUTION

We confirm that all the authors are experts in this research topic and have contributed to the planning, writing and reviewing of this paper.
